# Impact of reducing colistin use on colistin resistance in *Escherichia coli* isolated from humans and poultry in Indonesia (COINCIDE): A protocol for a multisectoral, transdisciplinary One Health study

**DOI:** 10.1016/j.onehlt.2026.101347

**Published:** 2026-01-30

**Authors:** Soe Yu Naing, Juliëtte Severin, Aldert Zomer, Kuntaman Kuntaman, Imron Suandy, Sunandar Sunandar, Annisa Rachmawati, Nofita Nurbiyanti, Mira Leonie Schneiders, Koen Peeters Grietens, Alicia Widya, Linda van der Graaf-van Bloois, Mathieu Pruvot, Diego Nobrega, Anis Karuniawati, Jaap A. Wagenaar

**Affiliations:** a Division of Infectious Diseases and Immunology, Department of Biomolecular Health Sciences, Faculty of Veterinary Medicine, Utrecht University, Utrecht, the Netherlands; b Department of Medical Microbiology and Infectious Diseases, Erasmus MC University Medical Center Rotterdam, Rotterdam, the Netherlands; c Department of Clinical Microbiology, Faculty of Medicine, Universitas Airlangga, Surabaya, Indonesia; d Dr. Soetomo General Academic Hospital, Surabaya, Indonesia; e Balai Besar Veteriner Denpasar, Denpasar, Indonesia; f Center for Indonesian Veterinary Analytical Studies, Bogor, Indonesia; g Department of Public Health, Institute of Tropical Medicine, Antwerp, Belgium; h Faculty of Veterinary Medicine, University of Calgary, Calgary, Alberta, Canada; i Department of Clinical Microbiology, Faculty of Medicine, Universitas Indonesia, Jakarta, Indonesia

## Abstract

**Introduction:**

Colistin is a last-resort antimicrobial used to treat infections caused by multidrug-resistant Gram-negative bacteria in humans. In Indonesia, widespread detection of colistin use and resistance in commensal *Escherichia coli* from poultry, particularly broilers, led to a national ban on its use in livestock effective since July 1st, 2020. However, the impact of this policy intervention on colistin resistance in both humans and livestock has not yet been evaluated. The COINCIDE study aims to investigate the ban's effect on colistin resistance, compliance, and transmission dynamics of resistance between humans and poultry.

**Methods and analysis:**

COINCIDE will: (i) assess phenotypic and genotypic colistin resistance in *E. coli* from humans and poultry; (ii) estimate transmission of colistin resistant *E. coli* between humans and animals; (iii) explore colistin and antimicrobial use (AMU) at the community level; (iv) identify social and cultural factors driving AMU; and (v) strengthen multisectoral One Health collaboration. Post-ban sampling will be conducted in three districts of Central Java Province (Klaten, Karanganyar, and Sukoharjo), where pre-ban samples were already available. We will recruit participants from primary healthcare centers (PHC) (*n* = 683), poultry farmers (*n* = 120), and visit a total of 60 small-scale layer farms. Broiler isolates (*n* = 2865) from Indonesia's routine antimicrobial resistance (AMR) surveillance (2018–2023) will also be included. In terms of sample collection, post-ban samples will include rectal swabs from patients visiting PHC and farmers and boot swabs from small-scale layer farms. Colistin resistance will be screened using CHROMagar COL-APSE agar medium and confirmed by broth microdilution. Results of long-read whole-genome sequencing will identify resistance mechanisms and transmission pathways. A qualitative ethnography work will include participant observation and informal conversations during field visits, alongside in-depth interviews with healthcare professionals and stakeholders. “Drug bag” method interviews will assess antimicrobial use, access, and drivers at the community level. Simulated patient visits to pharmacies and local shops (*warung* or *toko obat*), and interviews with agrovet outlets and poultry shops will be conducted to evaluate compliance with antimicrobial sales regulations.

**Conclusion:**

By integrating microbiological, epidemiological, policy, and social science data, findings from the COINCIDE study will provide a strong scientific basis to inform AMR policies in Indonesia, with potential significance to inform other countries across Southeast Asia.

## 1 Introduction

Antimicrobial resistance (AMR) remains a critical and growing threat to global public and animal health, economic stability, and health security. The 2024 high-level meeting on AMR at the United Nations General Assembly reaffirmed the continued political commitment to addressing this complex, cross-border One Health issue [1]. Since the adoption of the World Health Organization (WHO) Global Action Plan on AMR in 2015, member states have increasingly recognized the urgency of combating the AMR crisis and advanced their responses by developing and implementing national action plans on AMR (NAP-AMR). The Tracking AMR Country Self-Assessment Survey (TrACSS) reported a significant rise in NAP-AMR development, from 41% of member states in 2016–2017 to 88% by 2023–2024 [2]. Despite these efforts, AMR remains a significant burden, particularly in Southeast Asia, South Asia, and Sub-Saharan Africa [3]. Indonesia exemplifies this challenge, with its unique geographical and socioeconomic factors driving high antimicrobial use (AMU) in both humans and animals [4].

Indonesia is the world's fourth most populous country, consisting of over 17,000 islands, and approximately 285 million people [5]. Its large human population is complemented by substantial livestock populations: 3.71 billion poultry (mainly 84.9% broilers, 11.1% layers, followed by 4.0% free-range chickens), along with 4.11 million swine, and 11.75 million cattle [6]. Driven by population growth and rising demand for affordable protein, the broilers population alone was expected to grow from 3.28 billion head in 2024 to 3.4 billion head in 2025 [7], [8]. National initiatives such as the Makan Bergizi Gratis (MBG) program, which provides free nutritious meals to schoolchildren and pregnant women, further intensify food production pressures [9]. Meanwhile, outbreaks of infectious diseases including avian influenza, lumpy skin disease, African swine fever, and foot-and-mouth disease threaten livestock health and productivity [10]. Alarmingly, AMU in Indonesian livestock was projected to increase by 202% between 2010 and 2030 if current trends continue, highlighting the urgent need for effective strategies to curb AMR within a One Health framework [11].

**Fig. 1:**
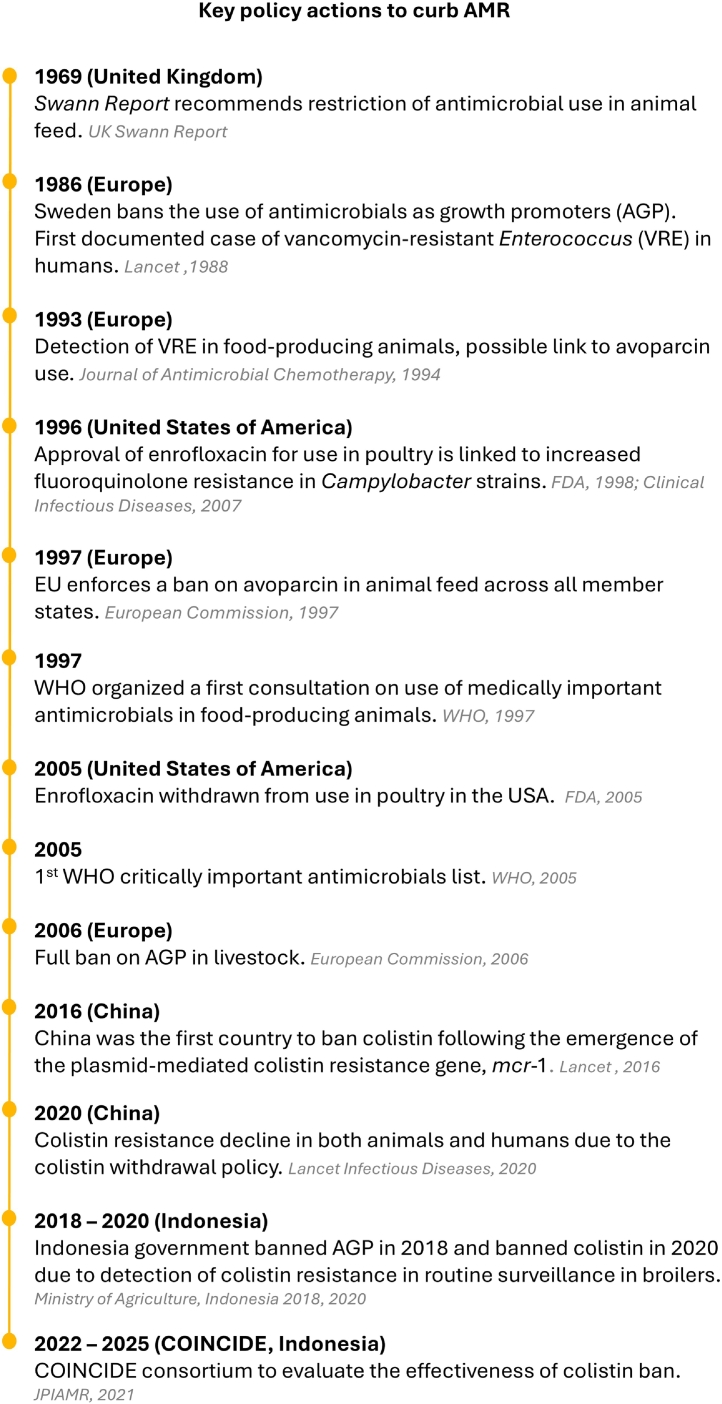
Roadmap showing key milestones in the withdrawal and restriction of antimicrobial use in animals through global policies and legislation, aimed at reducing antimicrobial resistance in humans and animals [13], [14], [18], [19], [20], [21], [22], [23], [24], [25].

One promising strategy to reduce AMR in One Health context is the withdrawal and restriction of antimicrobials used in animals through comprehensive policy and regulatory action [12]. Globally, regions such as Europe and North America have successfully reduced AMU and AMR by banning antimicrobial growth promoters (AGPs) and prophylactic use in livestock [13], [14]. In Indonesia, a key milestone was the 2017 NAP-AMR, confirming its commitment to advancing multisectoral One Health collaboration. Building on this NAP-AMR, the Indonesian government banned AGPs in 2018 under the Livestock and Health Law, which states “every person is prohibited from using feed ingredients mixed with certain hormones or antibiotics” [15]. More recently, in response to the detection of colistin use and colistin-resistant *E. coli* in healthy poultry through national AMR surveillance, Indonesian issued a ban on colistin use in livestock in 2020 [16], [17]. A summary of global and national interventions is presented in Fig. 1.

Colistin, also known as polymyxin E, is a last-resort antimicrobial critical for treating multidrug-resistant Gram-negative infections in humans. Although the clinical use of colistin in humans was limited in the 1980s due to toxicity concerns, the rise of carbapenem-resistant pathogens has renewed its importance [26]. The WHO classified colistin as a highest priority critically important antimicrobial (HP-CIA) for human medicine. However, in many Asian countries, including Indonesia, colistin has been widely used in livestock as a growth promoter and for prophylaxis, further contributing to driving colistin resistance development [27]. The discovery of plasmid-mediated mobilized colistin resistance (*mcr*) genes in 2016 transformed understanding of colistin resistance and AMR transmission, as these genes can spread rapidly between bacteria [28]. In response, countries such as China have restricted colistin use in agriculture, leading to measurable declines in resistance among both animals and humans [24]. Multiple studies from other countries including China, Japan, Portugal, and Spain also showed that banning or restricting colistin use in agriculture leads to a significant reduction in colistin resistance and *mcr*-1 gene prevalence among both animals and humans [29], [30], [31], [32], [33], [34], [35] (Table 1). In the case of Thailand where colistin was banned in 2017, Khine et al. (2022) reported that colistin-resistant *E. coli* prevalence in pigs dropped significantly from 60% in 2017 and 50% in 2018 to 3% in 2019, before rising again to 33% in 2020. In humans, colistin resistant *E. coli* were only detected in 2017 and disappeared in subsequent years [36]. The circulation of colistin resistance after the ban was observed in places like Thailand and it may be due to weak law enforcement and poor compliance with the colistin ban, as regulatory frameworks in Southeast Asia region are likely to be insufficient to ensure effective implementation [37].

**Table 1:** Prevalence change of *mcr* genes before colistin ban and colistin resistance across countries and sample types (MIC = minimum inhibitory concentration).

Country	Sample (Bacterial species)	Colistin resistance	Study period	Prevalence change (%)	Reference
China	Human hospital patients (*E. coli*)	*mcr-1*	2016–2019	14.3 → 6.3	[24]
China	Human community (*E. coli*)	MIC	2015–2019	1.7 → 1.3	
China	Pigs (*E. coli*)	MIC	2015–2018	34.0 → 5.1	
China	Chickens (*E. coli*)	MIC	2015–2018	18.1 → 5.0	
China	Pigs (*E. coli*)	*mcr-1*	2015–2019	38–45 → < 2	[29]
China	Broiler chickens (*E. coli*)	*mcr-1*	2016–2019	12.6 → 0.9	[35]
China	Pigs (*E. coli*)	*mcr-1*	2016–2018	45 → 19	[38]
China	Pigs (*E. coli*)	*mcr-1*	2017–2018	86.4 → 5.6	[34]
China	Human healthy volunteers (*E. coli*)	*mcr-1*	2018–2019	11.5 → 2.4	[30]
Japan	Pigs (*E. coli*)	*mcr-1*	2017–2018	17.8 → 6.7	[31]
Portugal	Poultry meat (Enterobacterales)	*mcr-1*	2020–2021	60 → 24	[33]
Spain	Healthy pigs (PCR only)	*mcr-1*	2017–2021	54 → 17	[32]
Thailand	Piglets and human (*E. coli*)	*mcr-1*	2017–2020	Piglets 60 (2017) → 3 (2019) → 33 (2020) Human 40 (2017) → 0 (2020)	[36]

**Fig. 2:**
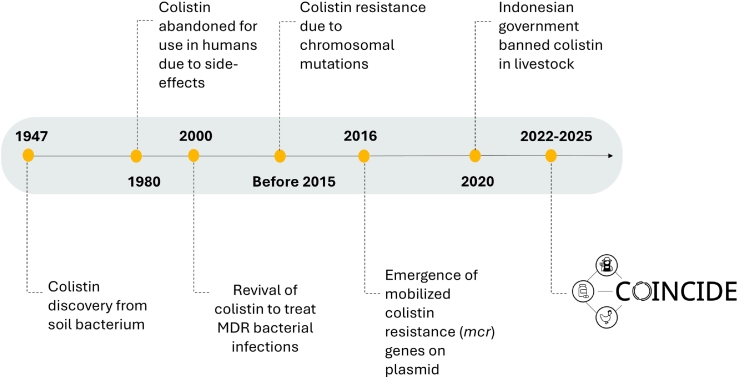
Timeline of colistin discovery, use, resistance, regulatory actions, and the origin of the COINCIDE consortium addressing colistin resistance (1947–2025). MDR, multidrug resistance.

Colistin was banned for use in livestock in Indonesia on July 1st, 2020. This policy ban provides a unique and timely opportunity to assess the effectiveness of such a policy in reducing AMR given its large poultry sector, prior colistin use and distinct regulatory and sociodemographic context (Fig. 2).

The COINCIDE study represents a transdisciplinary, multisectoral One Health collaboration involving medical doctors, microbiologists, veterinarians, epidemiologists, and social scientists, based at universities, research institutes, non-governmental organizations, and government bodies in Indonesia, Belgium, Canada, and the Netherlands. This consortium aims to evaluate the impact of the colistin ban on colistin resistance in *E. coli* from both poultry and human populations, integrating molecular epidemiology, microbiology, and social science research to understand the social and behavioral drivers of colistin and broader AMU and resistance.

## 2 Research objectives, scope and research questions

**Fig. 3:**
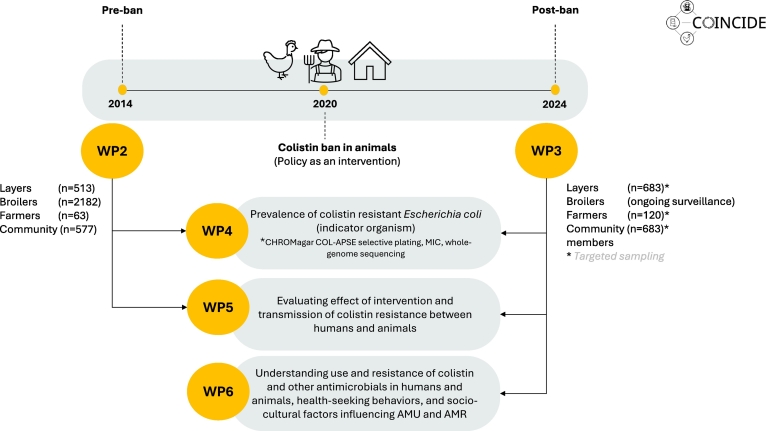
Overview of the workflow and COINCIDE work package themes, including details on the number of bacterial strains.

The COINCIDE study investigates the effect of Indonesia's colistin ban in livestock as an intervention to assess its impact on colistin resistance in *E. coli* from humans and poultry in Indonesia, as well as broader impacts associated with the ban. This project addresses this central question through four integrated perspectives: policy, sociocultural, epidemiological, and molecular approaches. The study scheme, consisting of work packages (WPs) aligned with specific research focus areas and their respective questions and methodologies within the multidisciplinary COINCIDE project, is shown in Fig. 3.

*E. coli* is used as an indicator organism in this study because it is commonly found in the intestines of humans and animals, is widespread in the environment, and serves as a key indicator in global AMR surveillance [39]*.* The first work package (WP1) includes coordination, and regulatory affairs. The pre-ban and post-ban sample collection (WP2 and WP3) focus on determining the prevalence of colistin-resistant *E. coli* before and after the ban by collecting and characterizing bacterial isolates from poultry (broiler and layer) and humans. Whole-genome sequencing (WGS) and bioinformatic analysis (WP4 and WP5) will explain resistance mechanisms, molecular epidemiology, and transmission pathways.

The social science work package (WP6) will contextualize epidemiological and molecular findings and investigate colistin and AMU within broader sociocultural contexts. WP6 examines how adherence to public health interventions and their effectiveness are shaped by social and cultural factors, including perceptions of drug efficacy and safety, illness severity, healthcare provider choice, gender and socioeconomic inequalities, historical experiences, and trust in health systems and government. Access-related factors such as drug availability and affordability further drive antimicrobial misuse. These socioecological determinants of AMU and AMR remain underexplored in Indonesia. Social science research is therefore essential to understand community-level drivers of broader AMU and colistin use among farmers, healthcare workers, and community members, as well as the acceptability of interventions, such as the colistin ban, and adherence to them.

Ultimately, this integrated approach aims to provide a comprehensive understanding of the impact of the colistin ban from multiple perspectives and critical evidence to guide future strategies for reducing AMU in livestock and humans in Indonesia.

## 3 Methods

### 3.1 Study design and setting

**Fig. 4:**
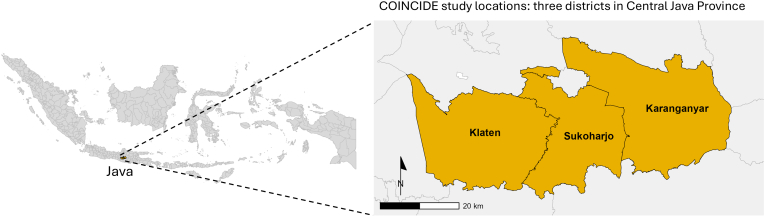
COINCIDE study location showing three districts: Klaten, Sukoharjo, and Klaten in the province of Central Java, Indonesia.

The COINCIDE study adopts a cross-sectional comparative design embedded within a One Health framework, supporting simultaneous assessment of colistin resistance and AMU across human and poultry sectors. Sampling will be conducted prospectively and compared with archived pre-ban data collected in 2014. Three districts in Central Java, Sukoharjo, Klaten, and Karanganyar are selected as study locations to maintain consistency with pre-ban data collection from layer farms in Central Java, Indonesia (Fig. 4). These districts provide a diverse social, cultural, and economic setting, where small-scale livestock farming, local health-seeking behaviors, and routine antimicrobials use create an ideal context for studying community- and farm-level antimicrobial practices.

### 3.2 Sample size and sampling strategies

To detect a meaningful reduction in colistin resistance from an estimated 15% before the ban to 10% afterward, power calculations indicated the need to analyze at least 683 *E. coli* isolates and risk factor surveys from both human and poultry sources. The retrospective (pre-ban) isolates include 577 archived human isolates from primary health care centers (PHC), 2182 broiler surveillance isolates, 513 layer chicken isolates, and 63 layer farmer isolates from 2014. Regarding the prospective sampling, human participants (*n* = 683) will be recruited from PHC, with eligibility criteria including adults over 18 years without current diarrhea or vomiting, who provide informed consent for rectal swab collection. Random selection via raffles of clinic attendees meeting these criteria will be used to minimize selection bias.

Up to 60 small-scale laying hen farms will be sampled to get an estimated 683 bacterial isolates, identified through local livestock office records. The small-scale layer farm is defined as having fewer than 10,000 layer chickens. Boot swabs will be collected from poultry houses. Farm workers (two or three from each farm, *n* = 120) will also be invited to provide rectal swab samples, enabling paired human-animal analysis. We will document recruitment and refusal rates in each setting to assess potential selection bias. All field teams will follow standard operating procedures developed by the COINCIDE consortium for sample labeling, cold-chain maintenance, and data recording to ensure quality and traceability. Additionally, available AMR data from the national broiler surveillance program from 2018 to 2023 will be incorporated. Within this surveillance program, approximately 800 caecal samples are collected from broilers per year via regional Disease Investigation Centers. From these samples, *E. coli* is isolated, and these isolates are tested for antimicrobial susceptibility including colistin using broth microdilution. These data provides a national perspective on resistance trends before and after the ban.

The complete COINCIDE sample scheme is also illustrated in Fig. 3.

### 3.3 Data collection

Data collection includes a range of methods, including biological sampling, quantitative surveys, qualitative interviews, ethnography field notes, participant observation, informal conversations, and in-depth interviews.

#### 3.3.1 Quantitative data

Human participants will complete questionnaires that document potential risk factors associated with the carriage of resistant bacteria, including demographics, dietary patterns, recent AMU, animal contact, access to water and sanitation, and medical history. Farmers will provide information on flock management, biosecurity measures, AMU in farm, vaccination, and disease history. Data collection tools, ODK digital surveys [40], will be developed in the local language (Javanese and Bahasa Indonesia) and will be collected by the local Indonesian research team and pre-tested with local research assistants for clarity and cultural appropriateness. Human rectal swabs and poultry boot samples will be transported under a cold chain to the laboratory for processing within 24 h.

#### 3.3.2 Qualitative data

Understanding the human behaviors and social factors driving AMU is critical in tackling AMR. COINCIDE integrates ethnographic approaches, including participant observation, informal conversations, in-depth interviews with key stakeholders (healthcare providers, pharmacists, technicians, public health workers, midwives, village cadres, farmers, veterinarians, and community members), and the participatory “drug bag” method interviews adapted from Dixon et al (2019) [41] to document household and farm antimicrobial usage, recognition and accessibility at community level. Simulated patient visits to pharmacies and local shops (*warung* or *toko obat*), along with interviews at agrovet shops will assess compliance with regulations governing antimicrobials, particularly colistin sales, that can reveal potential gaps in policy enforcement. Topic guides will be developed in an iterative manner. Qualitative data will be analyzed thematically and integrated with quantitative results to have a comprehensive understanding of macro-, meso-, and micro-level contextual factors influencing antimicrobial use and resistance in Indonesia.

### 3.4 Laboratory protocols

Laboratory protocols will be developed iteratively in collaboration with local laboratories, given options for capacity building and limited resources available. Human rectal swab and poultry/environmental boot swab samples will be cultured for *E. coli* on MacConkey agar. For human isolates, bacterial identification will be done using an automatic machine (VITEK® 2 COMPACT, bioMerieux). One presumptive *E. coli* colony per human sample and 15 *E. coli* isolates from boot swabs from layer farms will be selected. For animal isolates, *E. coli* isolates will be confirmed using biochemical tests. In parallel, isolates will be plated on CHROMagar™ COL-APSE (selective medium) in a tailor-made spot-test protocol that allows screening up to eight isolates per plate to screen for colistin-resistant growth. The protocol has been published on https://www.protocols.io, and the video protocol is available online (https://youtu.be/KC1x9u4d02g). Each batch of CHROMagar plates will include a colistin-susceptible *E. coli* strain (ATCC 25922) and a known *mcr*-1-positive *E. coli* as negative and positive controls, respectively. All isolates that grow on CHROMagar (presumptive colistin-resistant) will be tested using automated broth microdilution system (Sensititre, ThermoFisher) to determine colistin MIC, following the most recent EUCAST/CLSI standards. MIC results will be interpreted using the EUCAST MIC and ECOFF database. The ECOFF for colistin resistance in *E. coli* is ≤2 mg/L [42]. All isolates will be catalogued and stored at −80 °C in laboratories in Indonesia. Colistin-resistant *E. coli* isolates will be sequenced using Oxford Nanopore Technologies (ONT) long-read sequencing. DNA extraction, library preparation, and sequencing will be performed in-country by trained local staff. Where relevant, the Microbiology Investigation Criteria for Reporting Objectively (MICRO) framework will be followed in reporting [43].

### 3.5 Local capacity building

We will conduct bioinformatics analyses to identify resistance mechanisms, monitor molecular epidemiology (e.g., sequence typing), and trace transmission dynamics. These include genome assembly, annotation, resistance profiling, plasmid characterization, and phylogenetic reconstruction. The work will use command-line tools and public bioinformatics resources. To ensure sustainability and local ownership, we will implement a comprehensive capacity-building program that trains local microbiologists, technicians, government officials, and private sector partners in the format of installing of own computing capacity, hands-on training on locally generated data, workshops, peer-to-peer training sessions, and active discussions through online platforms throughout the project and beyond. This multisectoral approach will strengthen national response systems and equip stakeholders with the expertise needed to advance antimicrobial resistance surveillance in Indonesia.

### 3.6 Data management plan and data analysis

All data will be securely stored using the Yoda database and ODK data collection forms [40]. Personal identifiers will be pseudonymized or removed to preserve confidentiality. These data will only be available exclusively to principal investigators and data collectors. We will conduct periodic data audits and cleaning to ensure completeness and accuracy. Qualitative data will be analyzed using NVivo version 14. Genomics data will be analyzed using the command line and public bioinformatics resources, where available. All statistical analyses will be carried out using appropriate software such as R version 4.2.3, with a significance level of 0.05, adjusted for multiple testing, applied throughout unless otherwise specified.

### 3.7 Ethics approval and consent

Ethical approval for the 2014 pre-ban study was obtained with ethical certificate number LB.02.01/5.2/KE.011/2014, and it was part of the study conducted between 2013 and 2016. For the post-ban data collection, the study received ethical approval from the Faculty of Medicine, Universitas Indonesia – Cipto Mangunkusumo Hospital, Indonesia (number KET-294/UN2·F1/ETIK/PPM.00.02/2022); the National Research and Innovation Agency (BRIN), Indonesia (number B-2109/II/HM.00.01/4/2022, EC number: LB.02.01/5.2/KE.011/2014 for pre-ban data collection); the Institute of Tropical Medicine, Antwerp, Belgium (number 1652/22); and the Animal Care Committee at the University of Calgary, Canada (number AC22–0143). The local regional clearance approvals were obtained from the respective districts and sub-districts. The COINCIDE study is also registered at www.ClinicalTrials.gov (NCT number NCT05960084).

Informed consent will be obtained from all participants before any data or specimen collection, except in the case of drug sellers involved in simulated patient visits, where consent would compromise the validity of the approach. Participants will provide verbal informed consent for the use of photographs, participation in questionnaires, and interviews, including audio recordings, which will be transcribed and translated by trained local fieldworkers. Verbal informed consent is preferred in this study context, because the act of signing one's name can represent a reason for mistrust, thereby reducing the quality of the data collected. Verbal informed consent will be documented by the lead researcher present at the time by completing the consent form, with the research assistant acting as a witness to the consent. Consent materials and interviews will be administered in the local languages used in the community, primarily Javanese and Bahasa Indonesia. Trained fieldworkers fluent in the appropriate languages and dialects will conduct the consent process to ensure comprehension and voluntariness. All human samples will be collected non-invasively via rectal swabs. No direct sampling from animals will be performed; instead, the boot swab technique will be used to collect fecal material from the farm environment, avoiding direct contact with the animals. All data will be anonymized to ensure participant confidentiality.

All procedures comply with international ethical and biosafety standards for research involving human participants, including the Declaration of Helsinki, Council for International Organizations of Medical Sciences (CIOMS) guidelines, and applicable Indonesia and EU regulations. Where applicable, protocols will align with the Nagoya Protocol on Access and Benefit Sharing to ensure equitable use of genetic resources and associated traditional knowledge.

## 4 Conclusion and impact

**Fig. 5:**
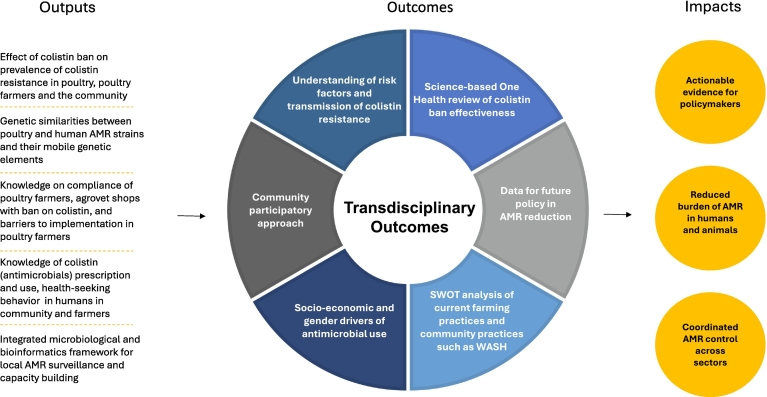
COINCIDE pathway to impact. SWOT means strengths, weaknesses, opportunities, and threats whereas WASH stands for water, sanitation and hygiene.

The protocol of the COINCIDE study is presented here with the aim of generating scientific evidence on the effectiveness of colistin restriction policies within the complex AMR landscape of Indonesia, as summarized in the COINCIDE impact pathway (Fig. 5). By integrating microbiological data from two One Health domains (humans and animals), molecular epidemiology, policy analysis, and social sciences, the project will provide a multidimensional understanding of the drivers and consequences of colistin use and resistance, as well as broader trends in AMU in Indonesia. The findings will support policymakers, health practitioners, livestock specialists, veterinarians, and the global scientific community in developing context-specific, evidence-based strategies to combat AMR. Furthermore, COINCIDE's interdisciplinary and locally grounded approach can serve as a scalable model for other countries in Asia facing similar AMR challenges across their human, animal, and environmental health sectors.

## CRediT authorship contribution statement

**Soe Yu Naing:** Writing – original draft, Writing – review & editing, Supervision, Visualization, Project administration, Methodology, Investigation, Conceptualization. **Juliëtte Severin:** Writing – review & editing, Supervision, Methodology, Investigation, Funding acquisition, Conceptualization. **Aldert Zomer:** Writing – review & editing, Supervision, Methodology, Investigation, Funding acquisition, Conceptualization. **Kuntaman Kuntaman:** Resources, Project administration, Methodology, Investigation. **Imron Suandy:** Resources, Project administration, Methodology, Investigation, Conceptualization. **Sunandar Sunandar:** Resources, Project administration, Methodology, Investigation, Funding acquisition, Conceptualization. **Annisa Rachmawati:** Resources, Project administration, Methodology, Investigation, Conceptualization. **Nofita Nurbiyanti:** Resources, Project administration, Methodology, Investigation, Conceptualization. **Mira Leonie Schneiders:** Writing – review & editing, Supervision, Resources, Project administration, Methodology, Conceptualization. **Koen Peeters Grietens:** Writing – review & editing, Supervision, Resources, Investigation, Conceptualization. **Alicia Widya:** Resources, Project administration. **Linda van der Graaf-van Bloois:** Project administration, Methodology. **Mathieu Pruvot:** Writing – review & editing, Methodology, Funding acquisition, Conceptualization. **Diego Nobrega:** Writing – review & editing. **Anis Karuniawati:** Writing – review & editing, Supervision, Resources, Project administration, Methodology, Investigation, Funding acquisition, Conceptualization. **Jaap A. Wagenaar:** Writing – review & editing, Supervision, Resources, Project administration, Methodology, Investigation, Funding acquisition, Conceptualization.

## Funding

The COINCIDE study is funded by the Joint Programming Initiative on Antimicrobial Resistance (JPIAMR)'s 2021 call on “One Health interventions to prevent or reduce the development and transmission of AMR”. The participating countries are supported by the following agencies: the 10.13039/501100000193International Development Research Centre (IDRC) for Indonesia (109860), 10.13039/100005622the Netherlands Organization for Health Research and Development (ZonMw) for the Netherlands (COINCIDE 10570132110006), the 10.13039/100005930Research Foundation, Flanders (FWO) for Belgium (G0G4921N), and the 10.13039/501100000024Canadian Institutes of Health Research (CIHR) for Canada (17893). The funders had no role in study design, data collection and analysis, decision to publish, or preparation of the manuscript.

## Declaration of competing interest

The authors declare that they have no competing interests.

## Data Availability

No data was used for the research described in the article.
